# LncRNAH19 improves insulin resistance in skeletal muscle by regulating heterogeneous nuclear ribonucleoprotein A1

**DOI:** 10.1186/s12964-020-00654-2

**Published:** 2020-10-28

**Authors:** Weiwei Gui, Wei Fen Zhu, Yiyi Zhu, Shengjie Tang, Fenping Zheng, Xueyao Yin, Xihua Lin, Hong Li

**Affiliations:** Department of Endocrinology, the Affiliated Sir Run Run Shaw Hospital, School of Medicine, Zhejiang University, 3 East Qingchun Road, Hangzhou, 310016 Zhejiang China

**Keywords:** Long non-coding RNA H19, Insulin resistant, Lipid metabolism, Skeletal muscle, hnRNPA1

## Abstract

**Background:**

Skeletal muscle is essential for glucose and lipid metabolism. Growing evidence reveals the importance of long non-coding RNAs (LncRNAs) in metabolism. This study aimed to investigate the function of LncRNA H19 (H19) in lipid metabolism of skeletal muscle and its potential mechanisms.

**Methods:**

Glucose tolerance, serum insulin and lipid content in serum and skeletal muscle were determined in control and H19-overexpressed db/db mice. Lipid metabolism was evaluated in H19-overexpressed or H19-silencing muscle cells by detecting lipid contents and mitochondria related functions. The underlying mechanisms were explored by RNA pull-down, mass spectrometry and RNA immunoprecipitation (RIP).

**Results:**

H19 was downregulated in skeletal muscle of db/db mice. H19 overexpression in db/db mice inhibited lipid ectopic deposition in skeletal muscle, meanwhile improved glucose intolerance and insulin resistance as compared with control db/db mice treated with ad-GFP. Furthermore, overexpression of H19 reversed FFA-induced lipid accumulation and increased cellular respiration in muscle cells, while H19 knockdown exhibited opposite effects in muscle cells. Mechanistically, H19 interacted with heterogeneous nuclear ribonucleoprotein (hnRNPA1) which was validated by RNA pulldown and RIP analysis, which increased translation of fatty acid oxidation closely related genes PGC1a and CPT1b.

**Conclusion:**

Our data suggest that overexpression of H19 ameliorates insulin resistance by reducing ectopic lipid accumulation in skeletal muscle. The possible underlying mechanisms are that overexpression of lncRNAH19 promotes fatty acids oxidation via targeting of hnRNPA1.

**Video abstract**

**Supplementary Information:**

**Supplementary information** accompanies this paper at 10.1186/s12964-020-00654-2.

## Background

Type 2 diabetes mellitus (T2DM) is one of the most common health problems, and is gradually increasing both in incidence and prevalence. In 2015, approximately 415 million people across the world had diabetes mellitus, and the International Diabetes Federation estimates the number of patients will reach 642 million in 2040 [1]. Given the mounting evidence that insulin resistance (IR) is one of the most important causes of T2DM [2–4], it is possible that cellular components involved in IR could provide promising therapeutic targets for T2DM. IR, characterized by hyperinsulinemia, can also induce hyperglycemia, dyslipidemia and hyperuricemia. Fortunately, several recent studies have revealed some new pathogenic mechanisms for IR and T2DM. Marycz et al. [5] found that dysregulation of adipose-derived mesenchymal stem cells (ASCs) including reduced mitochondrial biogenesis, limited fusion and abundance of autophagosomes and autolysosomes formation contributed to the occurrence and development of IR. Similarly, Alicka et al. [6] reported that impaired multipotency such as damaged proliferation, viability of ASCs which ultimately led to T2DM. In their findings, ASCs were a new promising therapeutic target for IR and T2DM. Interestingly, there were several articles identifying the potential role of metformin in repairing damaged ASCs in a way that decreased reactive oxygen species and promoted regenerations of ASCs [7, 8]. Nonetheless, the pathogenesis of IR and T2DM is complex and multifaceted and need more studies to uncover.

There is strong evidence indicating that skeletal muscle is the key tissue responsible for glucose disposal during insulin stimulation [9]. Under pathological conditions, skeletal muscle is insensitive to insulin and defective in its utilization of glucose, leading to IR in skeletal muscle, however the mechanism underlying this process is not well understood. Skeletal muscle IR is partly responsible for impaired fatty acid oxidation (FAO) and increasing influx of free fatty acid (FFA) [10]. As mitochondria are the major organelle for FAO in muscle cells, reduced mitochondrial contents and impaired mitochondrial function lead to decreased FFA utilization, which aggravates lipid overload and leads to IR [11].

Long non-coding RNAs (lncRNAs), which lack protein-coding capacity, are a class of transcripts over 200 nucleotides in length [12, 13]. Recently, a number of studies have uncovered a relationship between lncRNAs and metabolism. lncRNAs regulated in adipogenesis (lncRAPs) are required for timely maturation of adipocyte progenitor cells [14]. The lncRNA MIAT plays an essential role in the maintenance of the neuronal stem cell pool and terminal differentiation of neuron progenitors [15]. Overexpression of lncRNA TUG1 improves symptoms associated with diabetic nephropathy [16], and lncRNA SHGL suppresses hepatic gluconeogenesis and lipogenesis [17].

lncRNA H19, an important imprinted gene located on human chromosome 11, is abundantly expressed in embryogenesis, and decreases sharply with age in most tissues except skeletal muscle [18–20]. Intriguingly, there is decreased expression of H19 in the skeletal muscle of both T2DM patients and HFD-induced obese mice, and downregulation of H19 impairs glucose metabolism [21]. H19 has been shown to promote glucose metabolism via miR-106a-5p/E2F3 [22] and the PI3K-Akt signaling pathway [21]. However, previously reported roles of H19 in lipid metabolism have been controversial or even contradictory. Schmidt et al. [23] found that overexpression of H19 prevents the development of steatosis, whereas other studies showed that overexpression of H19 induces steatosis [24, 25]. Additionally, previous work revealed that H19 depletion impairs insulin sensitivity of muscle cells via let-7, which in turn inhibits key lipid metabolism genes [21]. Unfortunately, there are only a limited number of reports regarding this aspect of H19 function. Thus, these inconsistent findings require further investigation. As skeletal muscle is the main site fat conversion into energy, it is necessary to understand the specific function of skeletal muscle in lipid metabolism and develop new strategies for combating insulin resistance. However, much work is still required to identify the physiological role of H19 in lipid metabolism in skeletal muscle. To study the specific role of H19 in the pathology underlying insulin resistance, we used db/db mice as our model, which are widely used to study type 2 diabetes and obesity [26–28]. Our analysis further clarifies the role of H19 in lipid metabolism in skeletal muscle and its connection to insulin resistance.

## Materials and methods

### Experimental animals

All the experiments were approved by the Animal Care Committee of Zhejiang University. Male 6-week-old C57BL/6 J mice (*n* = 14), male C57BL/KsJ-db/m (db/m) (*n* = 6) and male C57BL/KsJ-db/db (db/db) mice (*n* = 12) were purchased from the Model Animal Research Center of Nanjing University (Nanjing, China), and were maintained on a 12 h light-darkness cycle in a specific-pathogen-free animal laboratory. After a week of adaptive feeding, mice were granted free access to water and received either a normal-chow diet (NCD) (*n* = 7) or high-fat diet (HFD, 35% carbohydrate, 20% protein and 45% fat) (*n* = 7) for 3 months under controlled light and temperature conditions in order to induce phenotypes associated with obesity and insulin resistance. After 3 months, the quadriceps femoris muscle was collected from each mouse.

Db/db mice were provided free access to water and NCD, and were randomly divided into control mice and H19-overexpression mice after one week of adaptive feeding. There were 6 mice in each group.

### Overexpression of H19 in db/db mice

The adenoviruses (Ad) expressing H19 and ad-green fluorescent protein (GFP) were constructed by Shanghai Genechem Company. To overexpress H19 in db/db mice, we injected 1.0 × 10^9^ plaque forming units of Ad-H19 or Ad-GFP into the tail vein once a week. After 2 weeks of treatment, oral glucose tolerance tests (OGTT) (1.0 g glucose/kg body weight) were carried out after an overnight fast, and tail blood was obtained to measure blood glucose levels using One Touch Ultra glucose stripes (LifeScan, PA, USA) at 0, 15, 30, 60 and 120 min. Two days later, the mice were sacrificed by cervical dislocation. Blood samples were obtained and serum was immediately frozen at − 80 °C for further tests. Tissues, including the quadriceps femoris muscle and liver, were carefully excised and weighed, and then stored at − 80 °C for follow-up experiments.

### Isolation of satellite cells and cell culture

Primary satellite cells were separated from the quadriceps femoris muscles of 7-day-old C57BL/6 J mice [29]. Briefly, dissected muscles were washed twice with Hanks balanced salt solution (HBSS), then collected into digestive medium consisting of 1% collagenase (Solarbio), HBSS and phosphate-buffered saline (PBS). Samples were minced with fine scissors and transferred to a 50 ml centrifuge tube with 20 ml digestive medium. The tube was then incubated in a standard humidified tissue culture incubator (37 °C, 5% CO_2_) for 45 min with two five-minute agitations during the incubation. The digestion was stopped using growth medium consisting of Ham’s F10 medium (Invitrogen) with 10% fetal bovine serum (FBS, Bio-rad), and the specimen was then filtered through a 100 μm cell strainer. The mixture obtained was precipitated by centrifugation at 1400 g for 10 min, and only the satellite cells were collected for further experiments. To separate the satellite cells from fibroblasts, we adopted the density centrifugation method developed by Yablonka-Reuveni and Nameroff [30]. Briefly, the cell suspension was transferred to a 15 ml Falcon tube that contained 20% colloidal polyvinylpyrrolidone coated silica (Percoll; Sigma) layered on top of a 60% Percoll cushion. The sample was centrifuged at 1800 g for 20 min, after which satellite cells could be obtained at the interface of the 20 and 60% Percoll layers.

For cell culture, satellite cells were grown in growth medium (Dulbecco’s modified Eagle’s medium (DMEM) containing 10% FBS and 100 IU/ml penicillin-streptomycin). When the cells reached 70–80% confluence, the growth medium was replaced with differentiation medium (DMEM containing 2% horse serum) for 5 days. Mouse C2C12 myoblasts (American Type Culture Collection, ATCC) were grown in growth medium (37 °C, 5% CO_2_). The differentiation method use for myoblasts was similar to that used for primary satellite cells.

### Differentiated primary satellite cells transfection and infection

Transfection of differentiated primary satellite cells was performed in a 6-well plate. For H19 and hnRNPA1 knockdown, small interfering RNAs were used. Briefly, 5 pmol of si-Con, si-H19 or si-hnRNPA1 was mixed with Lipofectamine 2000 transfection reagent (Thermo Fisher Scientific) and added to cells according to manufacturer’s instructions. For H19-overexpression experiments, differentiated primary satellite cells were infected with ad-GFP or ad-H19. For hnRNPA1-overexpression experiments, the hnRNPA1-GV208 overexpression plasmid (OE-hnRNPA1) and GFP-GV208 (OE-vector) were obtained from Shanghai GeneChem Co., Ltd. (Shanghai, China). Lipofectamine 2000 transfection reagent was used for hnRNPA1 overexpression experiments. At 48 h after transfection or infection, RNA and protein were extracted from the cells for further analysis. The siRNAs were designed by Ribo Biotechnology (Shanghai, China). The sequences of H19 siRNAs (three H19 siRNAs were constructed as a mixture) and hnRNPA1 siRNAs were as follows:

si-NC, 5′-CTCCAGGGAGGTGATAGGAG-3′.

si-H19–1, 5′-GCAGAATGGCACATAGAAA-3′.

si-H19–2, 5′-GGATCCAGCAAGAACAGAA-3′.

si-H19–3, 5′-GCAGTCATCCAGCCTTCTT-3′.

si-hnRNPA1–1, 5′-AGAAGACACTGAAGAACAT-3′.

si-hnRNPA1–2, 5′-CGATGAAGGGAGGAAACTT-3′.

### RNA extraction and RT-qPCR analysis

Total RNA was isolated from frozen quadriceps femoris muscle and liver samples or from differentiated primary satellite cells using TRIzol reagent (Invitrogen). A reverse transcription kit (Yeasen, China) was used to synthesize cDNA in a 20 μl reaction containing 1–5 μg of total RNA. A SYBR Green kit (Yeasen, China) was used to perform RT-qPCR in a 20 μl reaction containing 0.5–1 μl cDNA. Target mRNA levels were normalized to that of β-actin as an internal control and changes in expression were calculated using the formula 2^-△△Ct^. The primer sets used are shown in Supplementary Table 1.

### Western blot analysis

Differentiated primary satellite cells in 6- or 12-well plates were quickly lysed in situ in 1x SDS-sample buffer (200 μl/well for 6-well plates or 100 μl/well for 12-well plates), followed by denaturation. For muscle tissue samples, frozen samples (20 mg) were immediately ground on ice in 200 μl RIPA lysis buffer (Fdbio science, #FD008) in the presence of protease inhibitors (Solarbio, P0100). Samples were then homogenized in 5× SDS-sample buffer at a final concentration of 1×, followed by denaturation. Protein samples were run on a 10% SDS-polyacrylamide gel, and Western blot analysis was performed. Primary antibodies were diluted 1:1000, and included anti-phosphorylated (p) AMPK (Cell signaling technology 2535), anti-AMPK (Cell signaling technology 2532), anti-phosphorylated (p) ACC (Cell signaling technology 3661 L), anti-ACC (Cell signaling technology 3662), anti-SIRT1 (Abcam, ab110304), anti-CPT1b (Proteintech, 22,170–1-AP), anti-CD36 (Abclonal, A19016), anti-PGC1a (Abclonal, A12348), anti-OXPHOS (Abcam, ab110413) and anti-GAPDH (Abclonal, Ac001). GAPDH was used as a loading control. The bands on Western blots were analyzed using Image J.

### Differentiated primary satellite cells lipid measurements

Lipid measurements from differentiated primary satellite cells were performed using a triglyceride assay kit (Nanjing Jiancheng, China, A110) and ELISA kit(Renjie bio, China, RJ17089. Briefly, following H19 overexpression or silencing in differentiated primary satellite cells, free fatty acids (FFA) consisting of 15 mmol/l oleic acid and 15 mmol/l palmitate acid were added to the cells for 16 h. The final working concentration of FFA in the cells was 0.75 mmol/l. After incubation, cells were lysed with standard RIPA lysis buffer and then centrifuged at 12,000 g for 10 min at 4 °C. The supernatant was isolated and stored on ice. Triglyceride (TG) and diglyceride (DAG) levels were quantified using the previously mentioned kits according to the manufacturer’s protocols.

### Nile red staining

Differentiated primary satellite cells were pre-treated with free fatty acids as described above. Cells were then washed three times with PBS, fixed with 4% formaldehyde and stained for 15 min with 0.05 μg/ml Nile red solution (Solarbio, China, N8440) to visualize lipid droplets. Cell nuclei were counterstained with 496 diamidino-2-phenylindole (DAPI, Yeasen, China) and images were acquired using a fluorescent microscope (Zeiss Germany).

### Immunohistochemical staining

Immunohistochemistry staining was performed on the muscle samples of db/db mice. Skeletal muscles were fixed in 4% formaldehyde and cut into 5 μm thick sections. Some sections were stained with hematoxylin and eosin (H&E) and the remaining sections were stained with Oli Red O solutions (Nanjing Jiancheng, China), according to manufacturer’s guidelines, to determine the lipid content in the skeletal muscle samples. Histology images were obtained using a microscope with a digital camera attachment (Nikon, Japan).

### Mitochondrial respiration analysis

The XF Mito Stress Test Kit (Seahorse Bioscience) was used to measure mitochondrial respiration of myotubes on an XFe96 Extracellular Flux Analyzer. The concentrations of oligomycin, carbonyl cyanide-4 (trifluoromethoxy) phenylhydrazone (FCCP) and rotenone/antimycin used were 1.0, 1.0 and 0.5 μmol/l, respectively. The oxygen consumption rate (OCR) was recorded as recommended by the manufacturer. The respiration experiments were normalized to the number of cells.

### Transmission electron microscopy

Fixed differentiated primary satellite cells were washed within 0.1 M PBS, fixed with 1% buffered osmium tetroxide for 1 h, and then stained with aqueous 2% uranyl acetate. The samples were washed three times in water and dehydrated in increasing concentrations of ethanol (50, 70, 90 and 100%). The samples were then cut into ultrathin 0.5 μm sections with a Leica UC7 ultramicrotome. A Hitachi H-7100 transmission electron microscope (Hitachi-High Technologies Co., Shimbashi, Tokyo, Japan) was used to analyze the stained sections.

### RNA immunoprecipitation (RIP)

RIP was performed using a Magna RIPTM RNA-Binding Protein Immunoprecipitation Kit with minor modifications. Briefly, cell lysates from 2.0 × 10^7^ cells per RIP reaction were lysed in RIP Lysis Buffer, incubated on ice for 10 min and centrifuged for at 4 °C 15 min at 14000 g. Anti-hnRNPA1 or mouse IgG (5 μg) was added to 50 μl magnetic beads, and the samples were incubated at RT for 30 min with gentle rotation. Then supernatant protein was added into each antibody/bead reaction, and the samples were incubated at 4 °C overnight with gentle rotation. After incubation, RNA was extracted from the binding reaction with TRIzol and analyzed by RT-PCR.

### RNA pull-down assay and mass spectrometry

Pierce™ Magnetic RNA-Protein Pull-Down Kit was used to perform RNA pull-down experiments. Biotin-labeled RNA probes were synthesized by Tsingke and 100 pmol of biotinylated RNAs were used for pull-down experiments. Total protein extracts were obtained from C2C12 myoblast cells using standard lysis buffer (25 mmol/l Tris-HCl, pH 7.4, 150 mmol/l NaCl, 1% NP-40, 1 mmol/l EDTA, 5% glycerol). Protein extracts were precleared with streptavidin magnetic beads (Thermo scientific) and then incubated with 100 pmol of biotinylated RNAs for 1 h at 4 °C. Beads were washed with wash buffer and the proteins bound to the RNA were analyzed by SDS-polyacrylamide gel followed by silver staining and mass spectrometry.

### Statistical analysis

All data are shown in this study as mean ± standard deviation (SD). Statistical significance was determined using two-tailed Student’s t-test, with *P* values < 0.05 considered significant. Each in vitro experiment was conducted in triplicate.

## Results

### The expression level of H19 was reduced in obese and insulin resistant mice

**Fig. 1:**
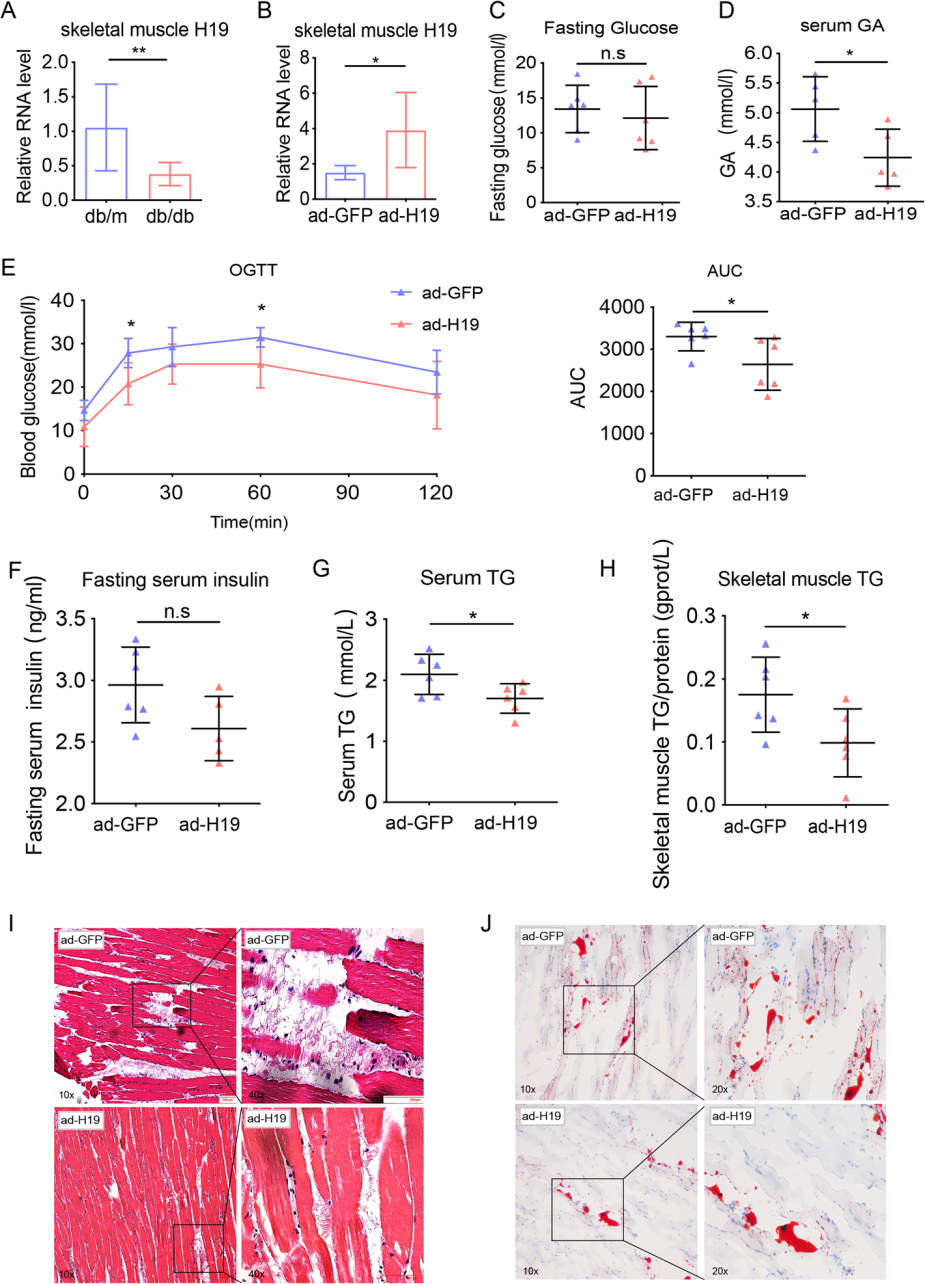
H19 overexpression in db/db mice ameliorates glucose intolerance and lipid ectopic accumulation. (**a**) The expression level of H19 was decreased in skeletal muscle of db/db mice. (**b**) Ad-H19 injection resulted in H19 overexpression in skeletal muscle of db/db mice. Fasting glucose levels (**c**) and serum GA levels (**d**) in db/db mice after H19 overexpression. (**e**) Left: OGTTs at 2 weeks after viral injection. Right: area under the curve (AUC) data. (**f**) Fasting serum insulin levels of db/db mice after H19 overexpression. Serum TG (**g**) and Skeletal muscle TG (**h**) levels in db/db mice after H19 overexpression. Representative images of Oil Red O staining assays (**i**) and H&E staining assays (**j**) of db/db mouse skeletal muscle after H19 overexpression. *n* = 6 mice per genotype. Numbers were the mean ± SD. *p < 0.05, **p < 0.01, n.s, not significant

The expression of H19 was reduced in skeletal muscle samples of the high-fat diet obese mice (Supplementary Fig. 1A). To test whether this could be recapitulated in the db/db mouse model, we examined H19 expression in skeletal muscle samples of db/db mice using RT-qPCR. As expected, db/db mice displayed reduced expression of H19 in skeletal muscle samples compared to control db/m mice (Fig. 1a).

### Overexpression of H19 ameliorated insulin resistance and glucose intolerance of db/db mice

To investigate the biological roles of H19 in glucose metabolism and its potential contributions to the development of obesity-induced insulin resistance, we used db/db mice as a model for obesity and insulin resistance. H19 overexpression in db/db mice was accomplished by tail vein injection of ad-H19 virus, followed by RT-qPCR to examine the effect of H19 overexpression in db/db mice. Using this method, we were able to achieve a 2.6-fold and 7.1-fold increase in H19 levels in skeletal muscle and liver, respectively(Fig. 1b and Supplementary Fig. 1B). We then assessed the effect of H19 overexpression on glucose metabolism by measuring multiple metabolic parameters. H19-overexpression in db/db mice had no effect on fasting glucose and body weight (Fig. 1c and Supplementary Fig. 1C). However, OGTT revealed that H19-overexpressing mice had significantly improved glucose tolerance (Fig. 1e), accompanied by a decrease in glycated albumin (GA) and fasting serum insulin levels (Fig. 1d and f). Collectively, these results indicate that elevated H19 levels ameliorate glucose intolerance and insulin resistance in db/db mice.

### H19 overexpression inhibited lipid accumulation in vivo and in vitro

Studies suggest that lipid accumulation in skeletal muscle impairs tissue-specific insulin sensitivity and ultimately leads to insulin resistance in the whole organism [31]. Subsequently, we explored whether H19 influences lipid metabolism in skeletal muscle. The levels of serum TG, serum cholesterol, liver TG and liver cholesterol were significantly lower in H19-overexpressing mice (Fig. 1g, supplementary Fig. 1D-F), indicating improvements in dyslipidemia and fatty liver symptoms. Additionally, skeletal muscle TG levels were diminished in H19-overexpressing mice (Fig. 1h). These effects were further validated using histological analysis and Oil Red O staining assay. Furthermore, ectopic lipid accumulation was reduced in skeletal muscle of H19-overexpressing mice compared to control mice treated with ad-GFP (Fig. 1i-j). Morphological observations indicated that H19 overexpression reduced hepatic lipid contents (Supplementary Fig. 1I). However, overexpression of H19 had no effect on ALT and AST, which are indicators of hepatic impairment (Supplementary Fig. 1G-H). Taken together, these data suggest that H19 overexpression alleviates excessive lipid deposition in db/db mice.

**Fig. 2:**
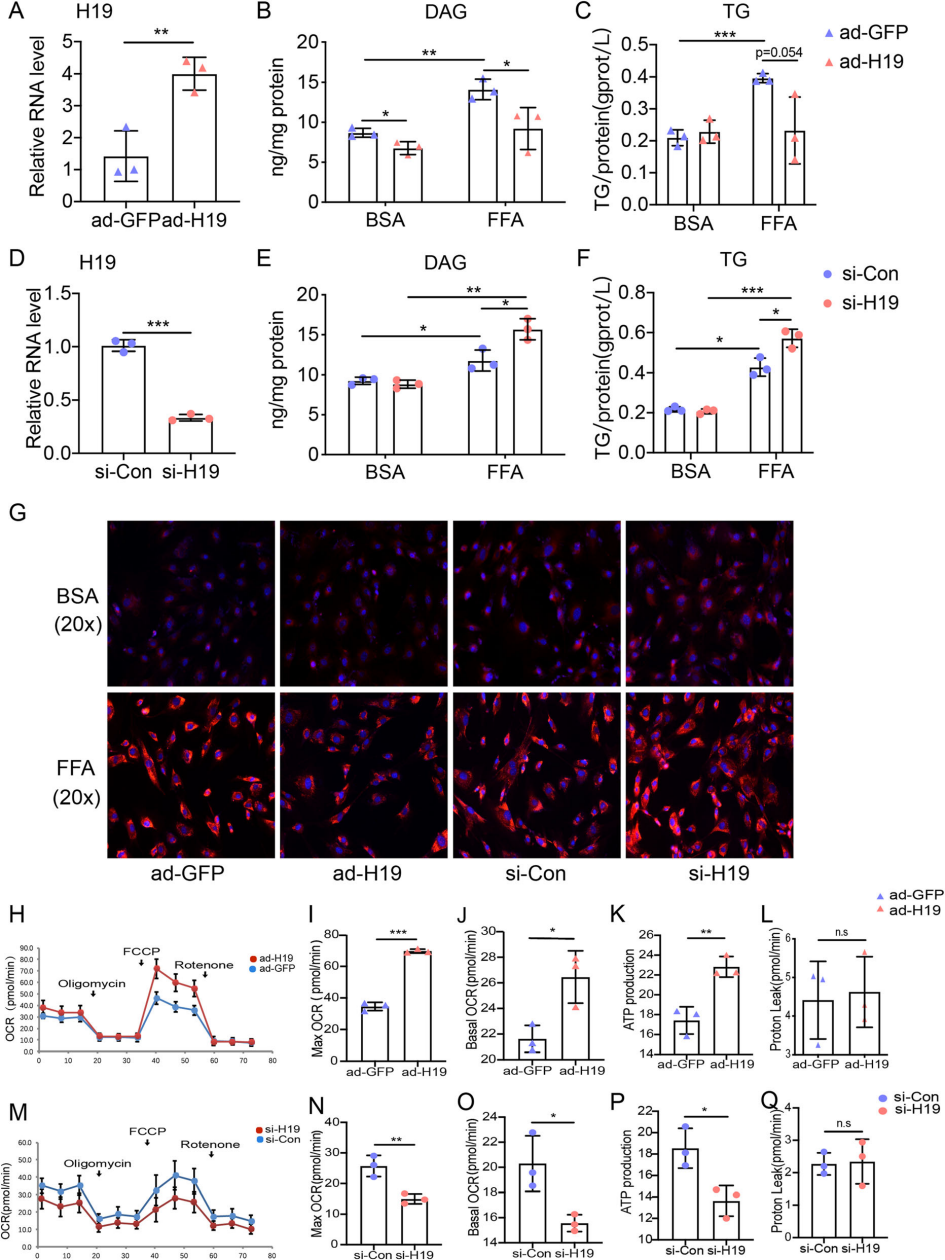
H19 regulates lipid metabolism in skeletal muscle cells. (**a**) Real-time PCR analysis of H19 expression in muscle cells treated with ad-H19. DAG contents(**b**) and TG contents (**c**)in muscle cells in the absence or presence of FFA after H19 overexpression. (**d**) Real-time PCR analysis of H19 expression in muscle cells treated with si-H19. DAG contents(**e**) and TG contents (F)in muscle cells in the absence or presence of FFA after H19 knockdown. (**g**) Representative images of Nile red staining assays in muscle cells of H19 overexpression or silencing. (**h**) Representative tracings of OCR measured in the H19 overexpression and control muscle cells. Maximal mitochondrial respiration (**i**), basal mitochondrial respiration (**j**), ATP production (**k**) and proton leak (**l**) of ad-H19 and ad-GFP infected muscle cells. (M) Representative tracings of OCR measured in the H19 knockdown and control muscle cells. Maximal mitochondrial respiration (**n**), basal mitochondrial respiration (**o**), ATP production (**p**) and proton leak (**q**) of si-H19 and si-Con transfected muscle cells. Quantification was based on 3 independent transfection/infection experiments. Numbers were the mean ± SD. ***p < 0.001, **p < 0.01, *p < 0.05

To assess the effects of H19 on lipid metabolism in vitro, quantitative lipid analysis was conducted on FFA-loaded extracts 48 h after transfection with si-H19 or infection of ad-H19. After infection with ad-H19, RT-qPCR analysis indicated that H19 expression significantly increased in differentiated primary satellite cells (Fig. 2a). We then transfected differentiated primary satellite cells with a mixture of three small-interfering RNAs targeted against H19, and using RT-qPCR, found that this effectively knocked down the expression of H19 (Fig. 2d). Intracellular TG and DAG were detected using the appropriate assay kits. In the presence of FFA, levels of DAG and TG in differentiated primary satellite cells decreased significantly following overexpression of H19, whereas no obvious effects were observed in the absence of FFA (Fig. 2b and c). Conversely, knockdown of H19 in differentiated primary satellite cells caused an increase in DAG and TG levels in the presence of FFA but no change in the absence of FFA (Fig. 2e and f).

Nile Red Staining was performed to further analyze the function of H19 in regulating the intracellular lipid content. The results showed that, in the presence of FFA, lipid levels were decreased in myotubes following H19 overexpression, whereas these levels increased following H19 depletion (Fig. 2g). Altogether, these data demonstrate that H19 overexpression inhibits lipid accumulation and H19 knockdown accelerates this accumulation.

### H19 overexpression promoted cellular respiration

Mitochondria are the main site of energy metabolism in the cell and play important roles in lipid metabolism. We next used an extracellular flux analyzer to investigate whether H19 affects lipid metabolism by regulating mitochondrial respiration. Both maximal and basal cellular respiration were significantly increased following H19 overexpression in differentiated primary satellite cells (Fig. 2h-j). H19 overexpression further affected cellular energy production by increasing the production of ATP in differentiated primary satellite cells (Fig. 2k). In contrast, depletion of H19 decreased both the maximal and basal respiration level, as well as ATP production (Fig. 2m-p). But there was no difference in proton leak after H19 overexpression or H19 knockdown in primary satellite cells (Fig. 2l and q). To further investigate whether H19 regulates mitochondrial biogenesis, we performed MitoTracker staining and mtDNA detection. We found that H19 overexpression or knockdown had no effects on mitochondrial contents (Supplementary Fig. 2A, C and D). We did, however, observe that the presence of FFA caused substantial defects in mitochondrial architecture, including perturbed cristae formation, in differentiated primary satellite cells, and these defects were abrogated by H19 overexpression (Supplementary Fig. 2B).

### H19 overexpression promoted expression of fatty acid oxidation-related genes

**Fig. 3:**
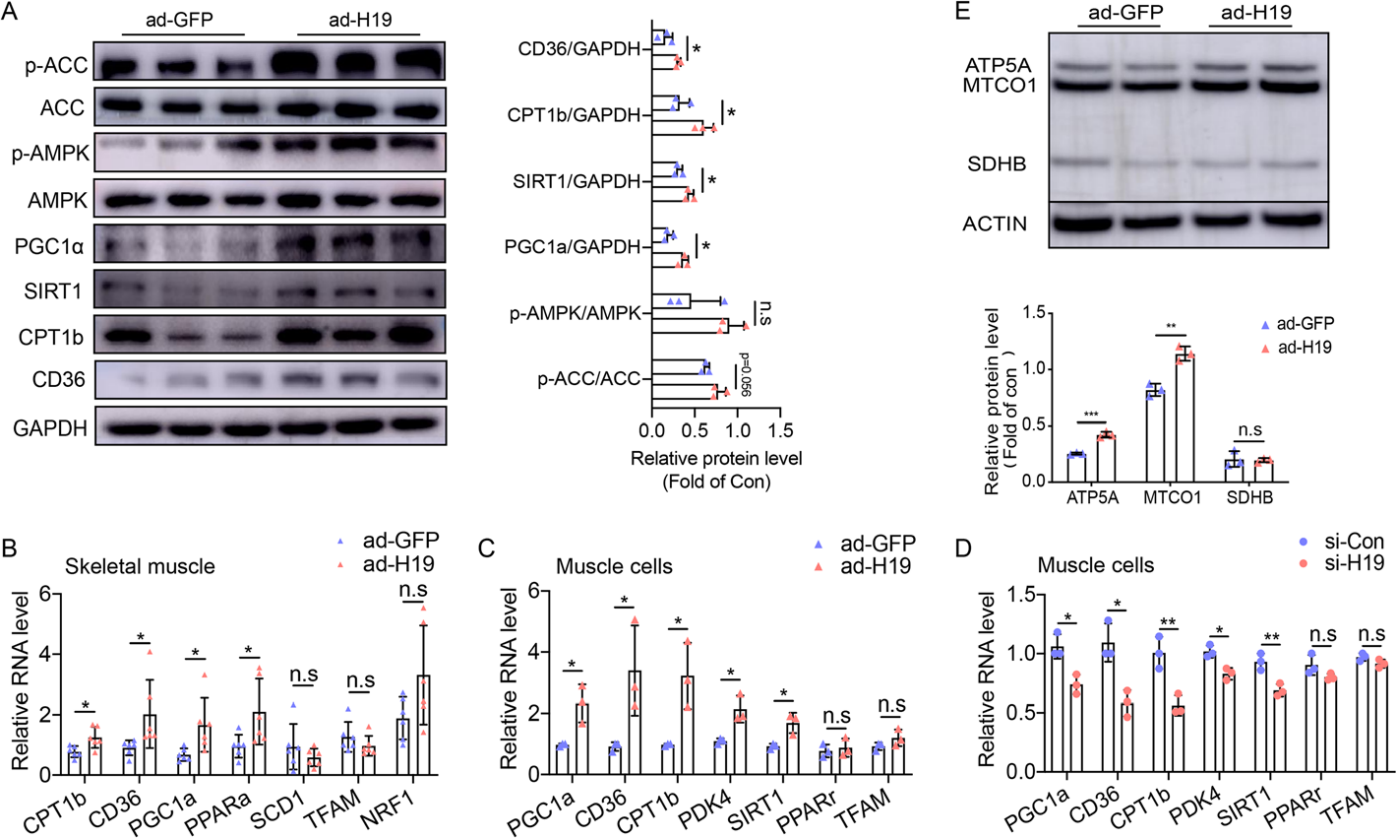
The effects of H19 on metabolic signaling pathway genes in skeletal muscles and cells. (**a**) H19 overexpression increased the protein levels of glucose and lipid metabolic genes in skeletal muscles. Representative gel images are presented in the left panel, and quantitative data are in the right panel. (**b**) H19 overexpression in skeletal muscles increased mRNA of glucose and lipid metabolic genes. H19 overexpression (**c**) and H19 knockdown (**d**) in muscle cells influenced mRNA of glucose and lipid metabolic genes. (**e**) H19 overexpression in muscle cells increased mitochondrial electronic chain complex proteins. *n* = 6 mice per genotype. Quantification was based on 3 independent experiments. Numbers were the mean ± SD. ***p < 0.001, **p < 0.01, *p < 0.05, n.s, not significant

Given that skeletal muscle plays important roles in fatty acid oxidation, which leads to changes in lipid content, we used RT-qPCR and western blot analysis to determine whether proteins in a signaling pathway related to fatty acid oxidation were altered in H19 overexpressing mice. H19 overexpression increased ACC and AMPK phosphorylation and the expression of PGC1a, SIRT1, CPT1b and CD36 in muscles (Fig. 3a). In addition, mRNA levels of CPT1b, CD36, PGC1a and PPARa in skeletal muscles were elevated after H19 overexpression, whereas there were no changes in the levels of SCD1, TFAM and NRF1 (Fig. 3b). Therefore, we next evaluated the expression of these genes in vitro. Similar to our results in skeletal muscle, the expression levels of PGC1a, CD36, CPT1b, PDK4 and SIRT1 increased in H19 overexpressing differentiated primary satellite cells and decreased in H19 knockdown cells (Fig. 3c and d). Furthermore, H19 overexpression led to increased expression levels of mitochondrial electronic chain complex proteins (ATP5A and MTCO1) (Fig. 3e).

### H19 recruited hnRNPA1 to regulate glucose and lipid metabolism

**Fig. 4:**
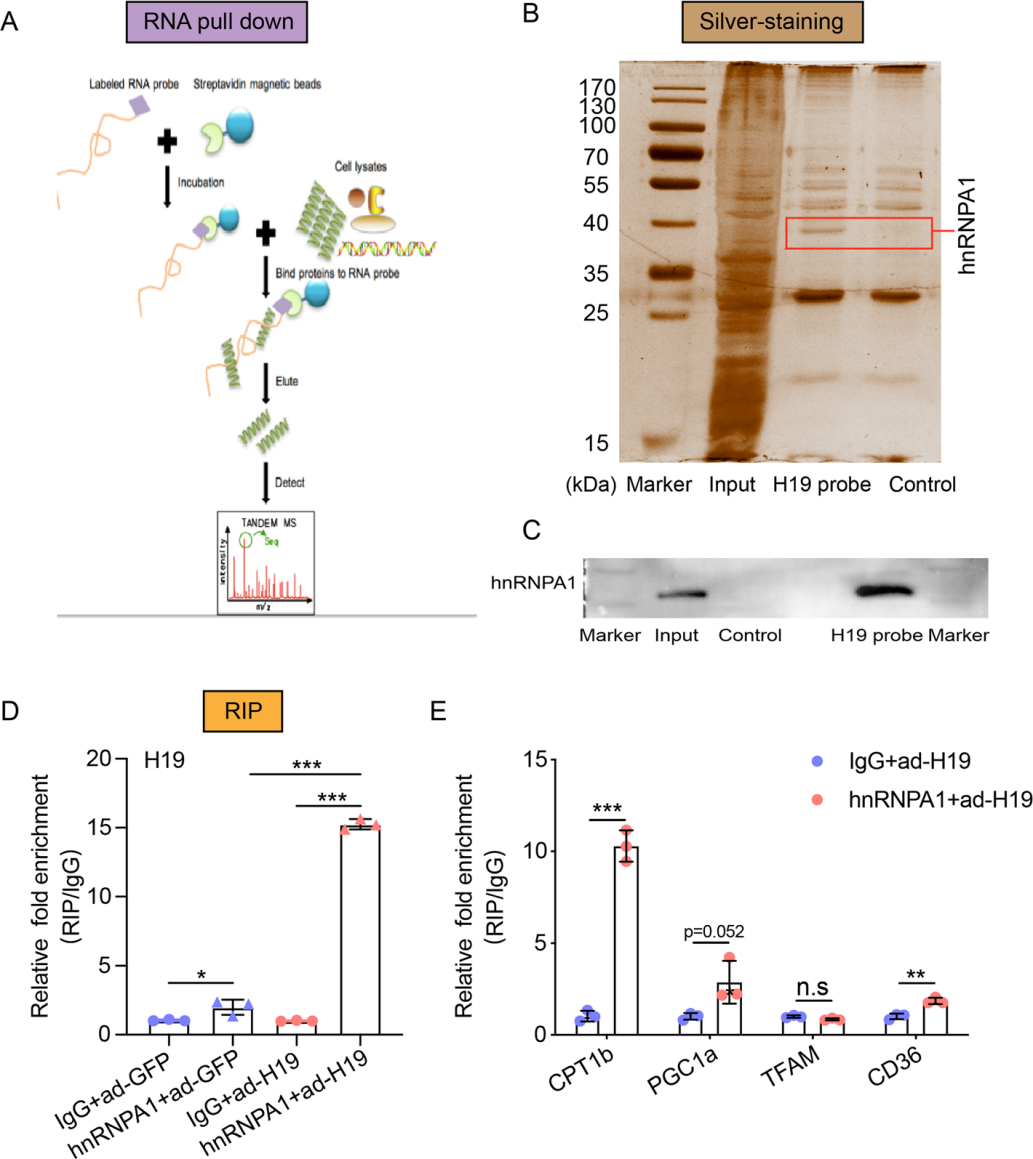
Validation of interaction between hnRNPA1 and H19. **a** Flow diagram of RNA pull-down assay. **b** Silver staining result of RNA pull-down assay revealed H19 interacted with hnRNPA1 in normal C2C12 cell lines. **c** Western blotting identified hnRNPA1 bound with H19. **d** RNA immunoprecipitation (RIP) revealed that hnRNPA1 was bound with H19. **e** RIP identified that hnRNPA1 was bound with CPT1b, CD36, PGC1a. Quantification was based on 3 independent transfection/infection experiments. Numbers were the mean ± SD. ***p < 0.001, **p < 0.01, *p < 0.05, n.s, not significant

LncRNAs typically regulate target genes via interactions with RNA binding proteins [32, 33]. To further determine the mechanism by which H19 regulates metabolism, we used a biotinylated H19 probe to perform an RNA pull-down assay, followed by silver staining and mass spectrometry (Fig. 4a and b). Through this analysis we identified the RNA binding protein hnRNPA1 as being bound to H19, and we confirmed this interaction by western blotting (Fig. 4c). Next, RNA immunoprecipitation (RIP) was performed in C2C12 cells using an hnRNPA1 antibody. H19 was present in the hnRNPA1 RIP sample at a much higher level than in the control IgG RIP sample. The increase was more apparent in H19 overexpression C2C12 cells, and was confirmed by RT-qPCR (Fig. 4d). We then used this RIP assay to explore target genes of hnRNPA1, and verified the association of hnRNPA1 with fatty acid oxidation-related genes such as CPT1b, PGC1a and CD36 (Fig. 4e).

**Fig. 5:**
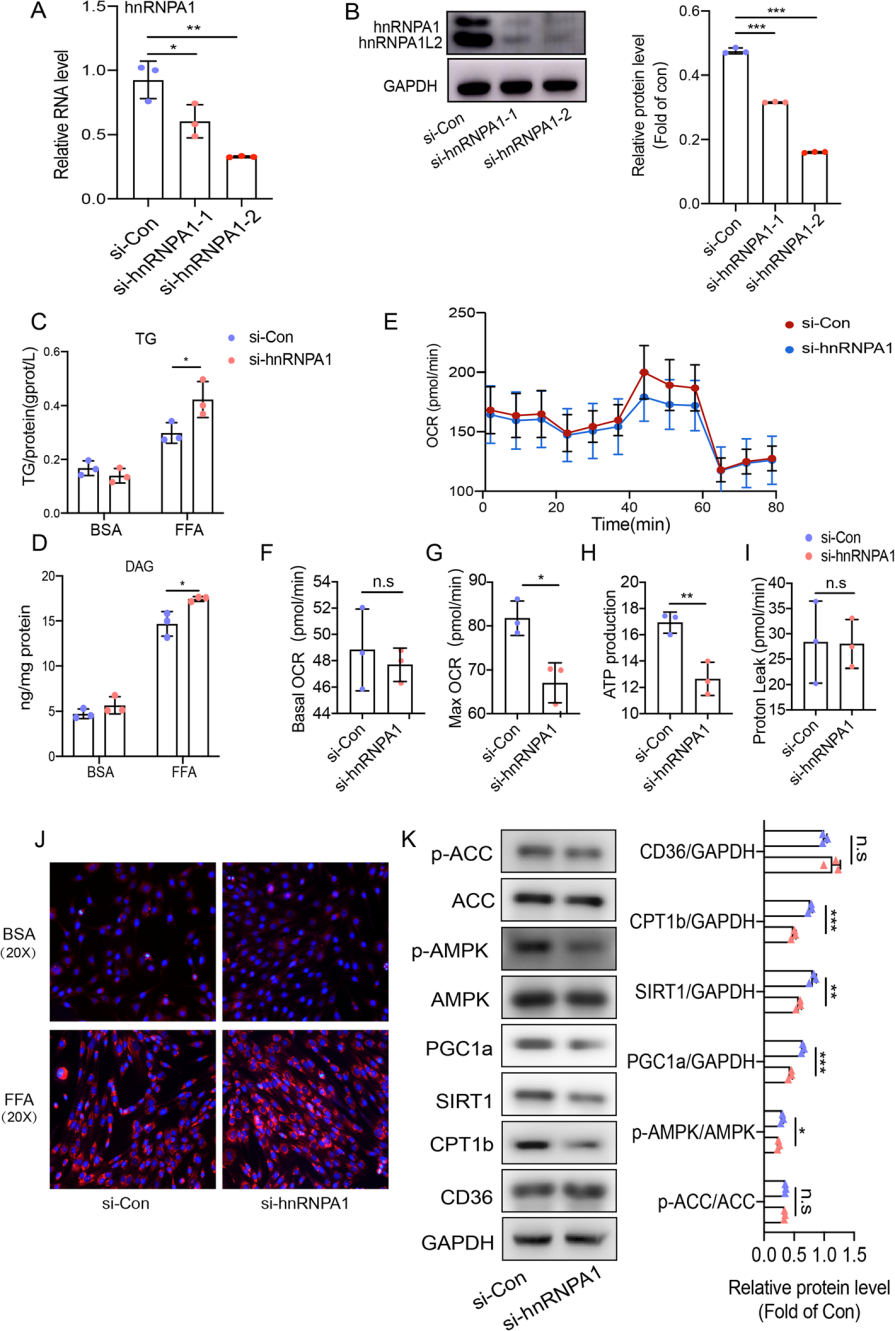
Silencing of hnRNPA1 stimulated lipid accumulation in muscle cells. (**a**) Real-time PCR analysis of hnRNPA1 expression in muscle cells treated with si-hnRNPA1. (**b**) Western blot analysis revealed that hnRNPA1 protein expression level was suppressed by si-hnRNPA1. Representative gel images are presented in the left panel, and quantitative data are in the right panel. TG contents(**c**) and DAG contents (**d**) in muscle cells in the absence or presence of FFA after hnRNPA1 knockdown. (**e**) Representative tracings of OCR measured in the hnRNPA1 knockdown and control muscle cells. Basal mitochondrial respiration (**f**), maximal mitochondrial respiration (**g**), ATP production (**h**) and proton leak (I) of si-hnRNPA1 and si-Con transfected muscle cells. (**j**) Representative images of Nile red staining assays in muscle cells of hnRNPA1 silencing. (**k**) hnRNPA1 knockdown decreased the protein levels of glucose and lipid metabolic genes in muscle cells. Representative gel images are presented in the left panel, and quantitative data are in the right panel. Quantification was based on 3 independent transfection/infection experiments. Numbers were the mean ± SD. ***p < 0.001, **p < 0.01, *p < 0.05, n.s, not significant

Next, we assessed the role of hnRNPA1 in lipid metabolism of differentiated primary satellite cells. We transfected differentiated primary satellite cells with two small-interfering RNAs targeting hnRNPA1, both of which successfully knocked down hnRNPA1 expression, as confirmed by qPCR and western blotting (Fig. 5a and b). si-hnRNPA1–2 was used in subsequent experiments. After transient hnRNPA1 knockdown, we analyzed intracellular lipid content and cellular respiration. As predicted based on our previous results, lipid quantitative assays revealed that hnRNPA1 knockdown increased FFA-induced lipid accumulation in muscle cells (Fig. 5c-d and j). Seahorse analysis revealed that maximal, but not basal, cellular respiration decreased significantly following hnRNPA1 knockdown in differentiated primary satellite cells (Fig. 5e-g). Additionally, hnRNPA1 knockdown further inhibited the production of ATP but had no effects on proton leak (Fig. 5h-i). Consistent with the findings described above, western blot analysis confirmed that hnRNPA1 knockdown decreased AMPK phosphorylation and the expression of PGC1a, SIRT1 and CPT1b, whereas no changes were observed in CD36 expression or ACC phosphorylation (Fig. 5k). HnRNPA1 knockdown exhibited the same effects as that of H19 knockdown in primary satellite cells which indicated that H19 might function by relying on hnRNPA1.

**Fig. 6:**
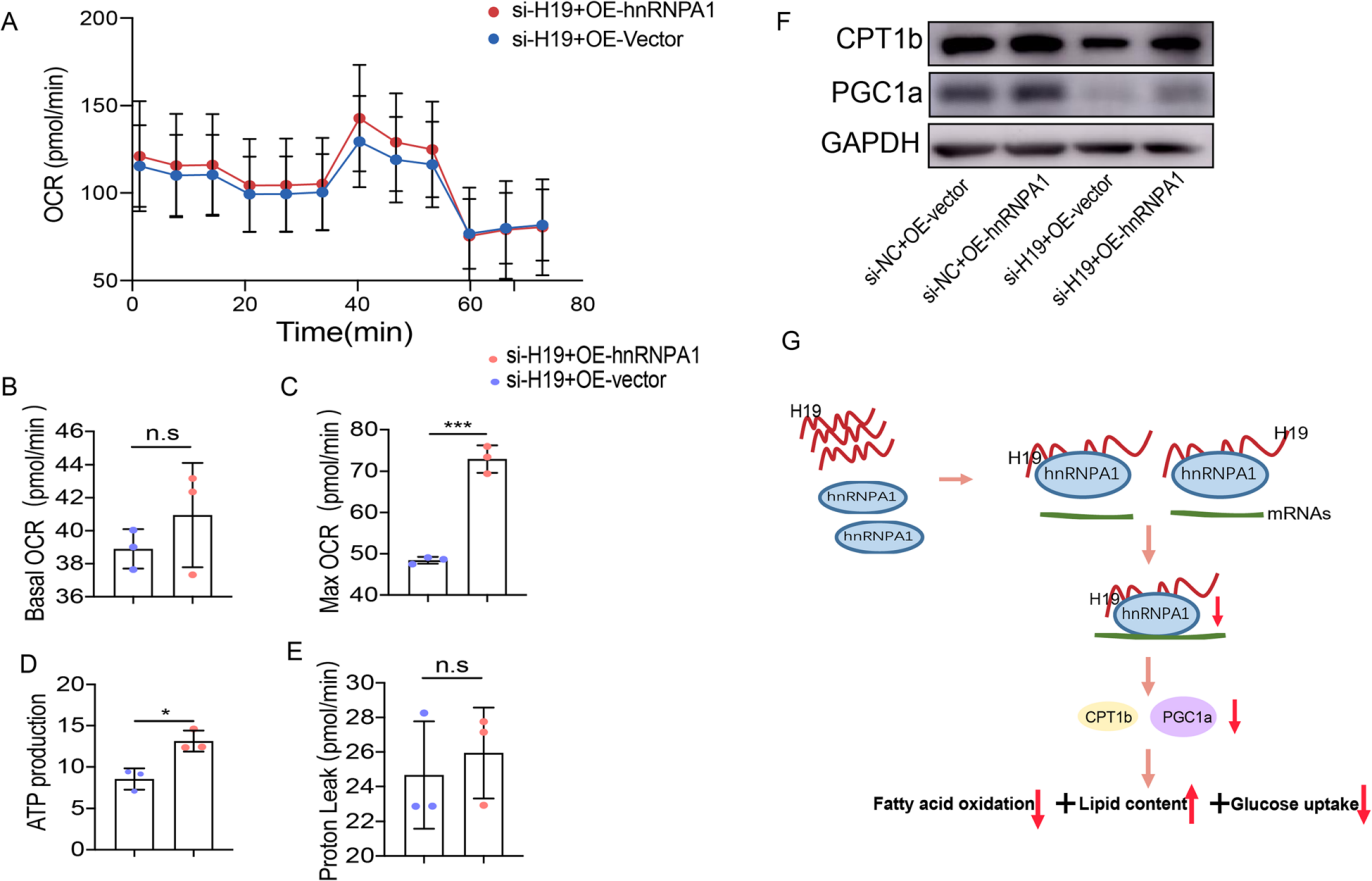
The effects of H19-hnRNPA1 complexes in metabolism. (**a**) Representative tracings of OCR measured in the hnRNPA1 overexpression and control muscle cells in the presence of H19 knockdown. Basal mitochondrial respiration (**b**), maximal mitochondrial respiration (**c**), ATP production (**d**) and proton leak (**e**) of OE-hnRNPA1 and OE-vector transfected muscle cells in the presence of H19 knockdown. (**f**) The protein levels of PGC1a and CPT1b of OE-hnRNPA1 and Vector transfected muscle cells in the presence of H19 knockdown. (**g**) Proposed model of H19-mediated regulation of lipid contents in skeletal muscle. Quantification was based on 3 independent transfection/infection experiments. Numbers were the mean ± SD. ***p < 0.001, *p < 0.05, n.s, not significant

In order to further assess the mechanisms by which H19 and hnRNPA1 affect metabolism, an hnRNPA1 overexpression plasmid (OE-hnRNPA1) and control vector (OE-vector) were constructed and transfected into si-H19-treated differentiated primary satellite cells. Cellular respiration and expression of fatty acid oxidation-related genes were analyzed in these cell lines. HnRNPA1 overexpression partially reversed inhibition of cellular respiration and ATP production induced by H19 knockdown (Fig. 6a-d) while didn’t influence proton leak in primary satellite cells (Fig. 6e). Furthermore, CPT1b and PGC1a protein levels were downregulated after H19 knockdown, and this decrease was partially rescued by hnRNPA1 overexpression (Fig. 6f). Taken together, our results suggest that H19-hnRNPA1 complexes play key roles in cellular lipid metabolism and mitochondrial functions in skeletal muscle by regulating the translation of fatty acid oxidation-related genes such as PGC1a and CPT1b (Fig. 6g).

## Discussion

LncRNAs are a class of transcripts with little or no protein-coding capacity. Although the functions of many lncRNAs are still unknown, there is growing evidence of the importance of lncRNAs in various biological processes, including embryology, metabolism and disease pathology [12, 13, 34]. H19 was first identified as an oncofetal lncRNA and is generally reported to play crucial roles in various types of cancer [35, 36]. Previous work has uncovered a role for H19 in glucose metabolism in skeletal muscle, and lipid metabolism in liver and adipose to some extent. Downregulation of H19 impaired glucose metabolism in skeletal muscle [21, 37]. Despite previous analysis, the role of H19 in lipid metabolism in the liver remains unclear. Furthermore, very few studies related to this topic have been reported. One study found that overexpression of H19 prevented the development of steatosis, however other studies have shown that overexpression of H19 induced steatosis [23–25]. We sought to explore the role of H19 in liver lipid metabolism and found that H19 overexpression in db/db mice ameliorated fatty liver symptoms. Surprisingly, our study also revealed an association between altered skeletal muscle lipid metabolism and H19. Skeletal muscle plays important role in lipid metabolism, however, the specific role of H19 in lipid metabolism in skeletal muscle, particularly its role in fatty acid oxidation and mitochondrial function, had not been previously analyzed. Therefore, it is necessary and valuable to explore the function of H19 in skeletal muscle lipid metabolism. Although tail vein injection of adenovirus in mice usually results in maximal delivery of the gene to the liver, several published studies observed phenotypes in skeletal muscle after tail vein injection [38–40]. Given this, we were able to focus on the effects of H19 in skeletal muscle lipid metabolism. We found that H19 was downregulated in muscle of both db/db mice and HFD-induced obese mice. Up-regulation of H19 ameliorated glucose intolerance and insulin resistance of db/db mice, and was accompanied by amelioration of ectopic lipid accumulation in skeletal muscle and liver.

Imbalance between rates of fatty acid uptake and fatty acid oxidation in muscle cells leads to an increase in intramyocelluar lipid contents. Mitochondria are the primary site of fatty acid oxidation in skeletal muscle. Free fatty acids taken up into muscle cells via passive diffusion transport proteins in the cell membrane (CD36) are then transformed into long-chain fatty acyl-coenzyme A (LCACoA). LCACoA is then taken up into mitochondria via carnitine-palmitoyltransferase 1 (CPT1) in the mitochondrial membrane, and undergoes β-oxidation there [41]. Mitochondrial dysfunction, including defects in oxidative capacity and reduced mitochondrial contents, impairs fatty acid oxidation and consequently causes fatty acid overload in muscles [42]. Thus, mitochondrial dysfunction partly accounts for lipid accumulation and can be a predisposing factor for insulin resistance [43, 44]. Our data demonstrates that up-regulation of H19 promoted cellular respiration and lipid oxidation, and reduced lipid accumulation in primary muscle cells without effecting mitochondrial biogenesis. H19 knockdown in primary muscle cells induced the opposite results, which further verified the role of H19 in lipid metabolism. We analyzed mitochondrial DNA copy number and performed MitoTracker staining, both of which revealed that H19 had no effect on mitochondrial contents. This is inconsistent with other studies, as it has been reported that H19 regulates brown adipose and skeletal muscle mitochondrial biogenesis [23, 37]. This discrepancy may be due to the use of different cell types and different methods. We used primary muscle cells while Schmidt et al. used primary adipocytes. Additionally, we up-regulated H19 specifically in muscle cells, whereas Geng et al. knocked down H19 in the whole mouse. Furthermore, in our study, upregulation of H19 activated ACC and AMPK, and promoted the expression of the fatty acid oxidation-related genes CPT1b and PGC1a.

LncRNAs usually regulate target genes by interacting with RNA-binding proteins (RBPs). An increasing number of studies have identified the important roles of RBPs in regulating the lncRNAs and their downstream target genes [24, 45–49]. We have identified hnRNPA1 as an interacting partner of H19 using RNA pull down and RIP assays. HnRNPA1 is reported to play many roles in regulating major steps in RNA metabolism, including transcription, splicing, stabilization and translation of transcripts. hnRNPA1 negatively regulates the transcription of thymidine kinase (TK) [50] and γ-fibrinogen [51], whereas it positively regulates the transcription of ApoE [52]. Many studies have reported the roles of hnRNPA1 in alternative splicing [53–55]. Recently, a number of studies have revealed important roles for hnRNPA1 in several biological processes, including glucose and lipid metabolism, mitochondria function and tumorigenesis. Here, hnRNPA1 was identified as a positive regulator of lipid metabolism in muscle cells, as it is able to regulate several key genes involved in fatty acid oxidation, such as CPT1b and PGC1a. Our results support a model whereby H19 recruits hnRNPA1 to CPT1b and PGC1a transcripts to modulate their translation during lipid metabolism. Therefore, our analysis revealed a potential new complex, H19-hnRNPA1, and a new pathway, H19-hnRNPA1-CPT1b/PGC1a, neither of which had been previously reported. We cannot, however, exclude the possibility of other unidentified factors participating in the regulation of H19, hnRNPA1, PGC1a and CPT1b, which need further investigations. Our work sheds new light on the mechanism of H19 in lipid metabolism and also provides a new candidate therapeutic target for treating diabetes mellitus.

However, there are some limitations in our study. We used tail vein injection of adenovirus to overexpress H19 in db/db mice. Thus, we cannot exclude the effects of H19 in the liver, although the liver phenotypes observed in our study were consistent with those previously reported [23]. For further confirmation of the specific role of H19 in skeletal muscle, we repeated the same experiments in vitro using primary skeletal muscle cells. Additionally, we did not set up normal mice as the control for db/db mice. However, db/db mice are frequently used as a mouse model for diabetes combined with insulin resistance.

## Conclusions

In summary, we found that the expression level of H19 was downregulated in the skeletal muscle of obese and diabetic mice, and up-regulation of H19 ameliorated insulin resistance and glucose intolerance due to its ability to promote fatty acid oxidation and reduce ectopic lipid accumulation. Conversely, down-regulation of H19 inhibited fatty acid oxidation and increased ectopic lipid accumulation. Furthermore, we identify hnRNPA1 as an interacting partner of H19, and we show that H19 functions in lipid metabolism through interaction with hnRNPA1 to increase the translation of fatty acid oxidation-related genes, including CPT1b and PGC1a.

## Supplementary Information

**Additional file 1: Supplementary figure 1.** (A) The expression level of H19 was decreased in skeletal muscle of HFD mice. (B) Ad-H19 injection resulted in H19 overexpression in liver of db/db mice. Body weight (C), serum cholesterol levels (D), liver TG (E), liver cholesterol (F), ALT (G) and AST (H) in db/db mice after H19 overexpression. (I) Representative images of morphological assay of db/db mouse livers after H19 overexpression. *n* = 6 mice per genotype. Numbers were the mean ± SD. ***p < 0.001, **p < 0.01, *p < 0.05, n.s, not significant.

**Additional file 2: Supplementary figure 2.** (A) Representative images of mitotracker staining assays in muscle cells of H19 overexpression or knockdown. (B) Representative electron microscopy images from mitochondria of C2C12 cells in the absence or presence of FFA after H19 overexpression. (C) Relative mitochondrial DNA copy numbers from skeletal muscles of db/db mice after H19 overexpression. (D) Relative mitochondrial DNA copy numbers in muscle cells of H19 overexpression or knockdown. n = 6 mice per genotype. Quantification was based on 3 independent transfection/infection experiments. Numbers were the mean ± SD. n.s, not significant.

**Additional file 3: Supplementary table 1.** Primer sequences used in this study.

## Data Availability

All data generated or analyzed during this study are included in this article.

## Abbreviations

ACC: Acetyl -CoA carboxylase
ALT: Alanine aminotransferase
AMPK: Adenosine 5′-monophosphate (AMP)-activated protein kinase
AST: Aspartate aminotransferase
ATP: Adenosine triphosphate
CD36: Cluster of differentiation 36
CPT1b: Carnitine-palmitoyltransferase 1b
DAG: Diacylglycerol
FAO: Fatty acid oxidation
FCCP: Carbonyl cyanide-4 (trifluoromethoxy) phenylhydrazone
FFA: Free fatty acid
GA: Glycated albumin
hnRNPA1: heterogeneous nuclear ribonucleoprotein A1
IR: Insulin resistance
LncRNAs: Long non-coding RNAs
MTCO1: Mitochondrially encoded cytochrome c oxidase I
mtDNA: mitochondrial DNA
NRF1: Nuclear respiratory factor 1
OCR: Oxygen consumption rate
OGTT: Oral glucose tolerance tests
OXPHOS: Oxidative phosphorylation
PDK4: Pyruvate dehydrogenase kinase 4
PGC1a: Peroxisome proliferator activated receptor γ coactivator 1 α
PPARa: Peroxisome proliferators-activated receptor α
RIP: RNA Immunoprecipitation
SCD1: Stearoyl-CoA desaturase 1
SIRT1: Sirtuin 1
T2DM: Type 2 diabetes mellitus
TFAM: Mitochondrial transcription factor A
TG: Triglyceride
TK: Thymidine kinase
