# Supplementary figures and images for: LncRNAH19 improves insulin resistance in skeletal muscle by regulating heterogeneous nuclear ribonucleoprotein A1
Source: Cell Commun Signal. 2020 Oct 28;18:173. doi: 10.1186/s12964-020-00654-2 (PMC7592379; doi:10.1186/s12964-020-00654-2)

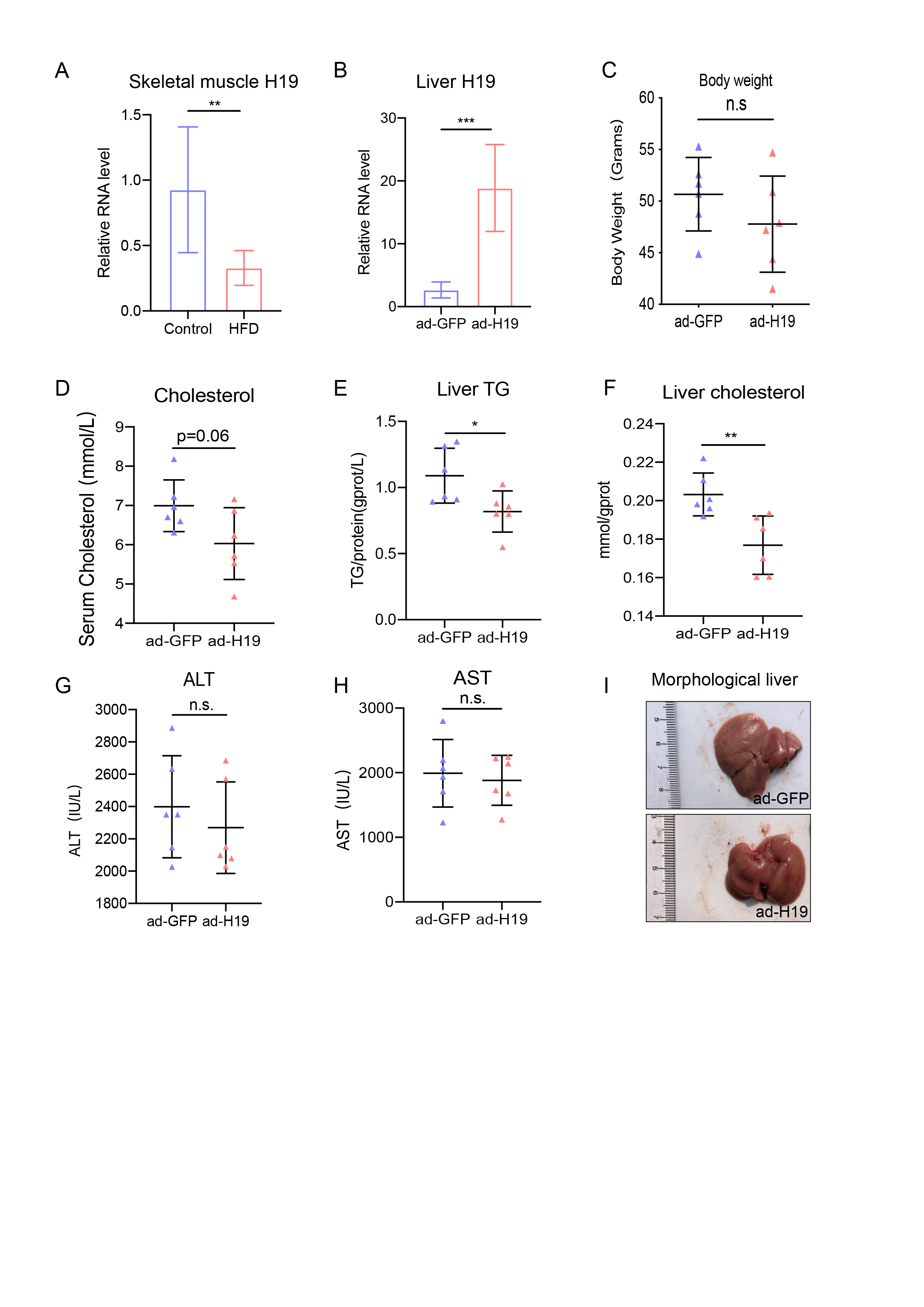

Supplement: Supplementary file 2 — Additional file 1: Supplementary figure 1. (A) The expression level of H19 was decreased in skeletal muscle of HFD mice. (B) Ad-H19 injection resulted in H19 overexpression in liver of db/db mice. Body weight (C), serum cholesterol levels (D), liver TG (E), liver cholesterol (F), ALT (G) and AST (H) in db/db mice after H19 overexpression. (I) Representative images of morphological assay of db/db mouse livers after H19 overexpression. n = 6 mice per genotype. Numbers were the mean ± SD. ***p < 0.001, **p < 0.01, *p < 0.05, n.s, not significant. [file 12964_2020_654_MOESM2_ESM.png]

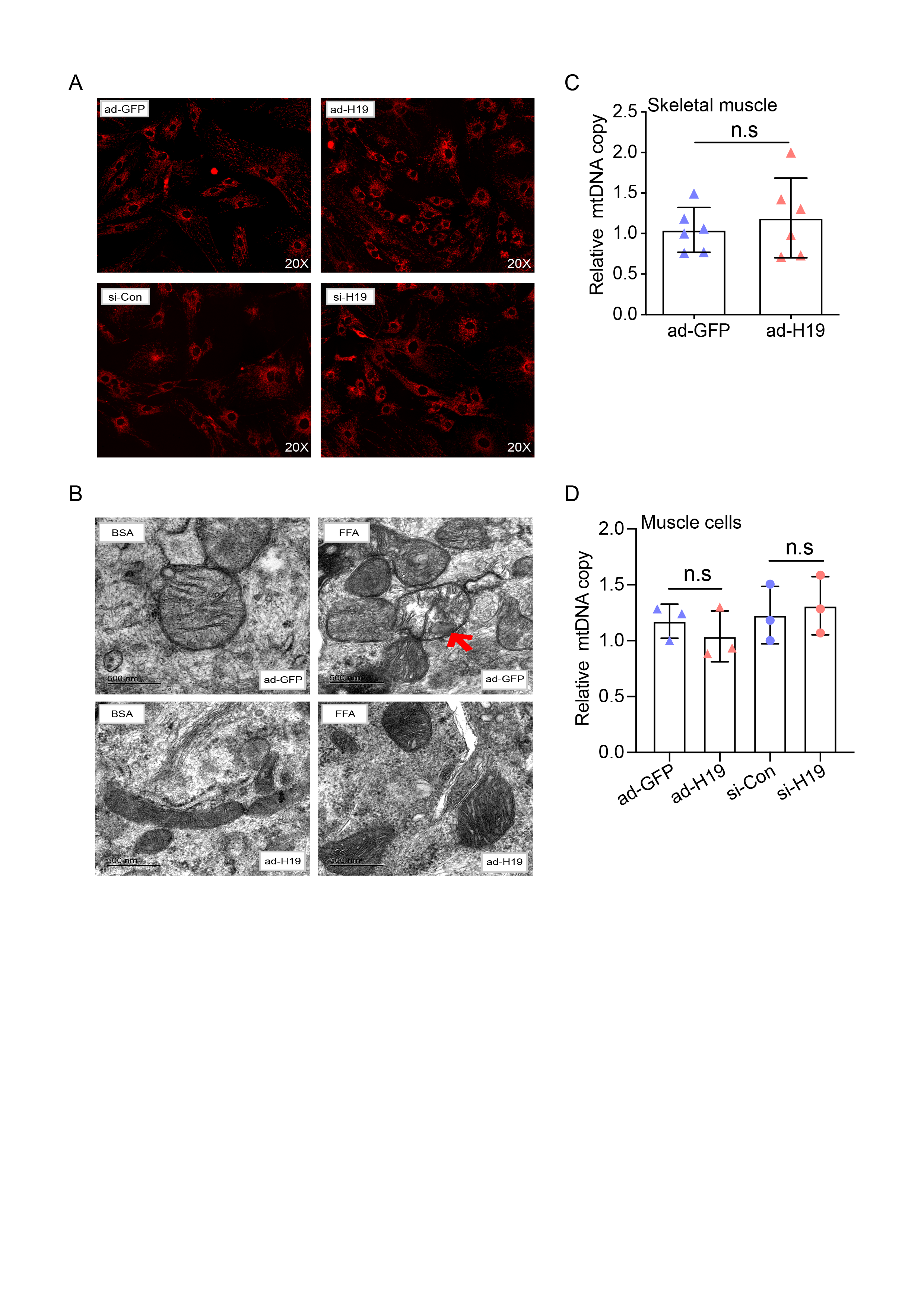

Supplement: Supplementary file 3 — Additional file 2: Supplementary figure 2. (A) Representative images of mitotracker staining assays in muscle cells of H19 overexpression or knockdown. (B) Representative electron microscopy images from mitochondria of C2C12 cells in the absence or presence of FFA after H19 overexpression. (C) Relative mitochondrial DNA copy numbers from skeletal muscles of db/db mice after H19 overexpression. (D) Relative mitochondrial DNA copy numbers in muscle cells of H19 overexpression or knockdown. n = 6 mice per genotype. Quantification was based on 3 independent transfection/infection experiments. Numbers were the mean ± SD. n.s, not significant. [file 12964_2020_654_MOESM3_ESM.png]
