# Supplementary material for: LncRNAH19 improves insulin resistance in skeletal muscle by regulating heterogeneous nuclear ribonucleoprotein A1
Source: Cell Commun Signal. 2020 Oct 28;18:173. doi: 10.1186/s12964-020-00654-2 (PMC7592379; doi:10.1186/s12964-020-00654-2)
Supplement: Supplementary file 4 — Additional file 3: Supplementary table 1. Primer sequences used in this study. [file 12964_2020_654_MOESM4_ESM.docx]

Supplementary table 1- Primer sequences used in this study.

|  | Sequences (5' to 3') |  |
| --- | --- | --- |
|  | sense | anti-sense |
| H19 | TGACTTCATCATCTCCCTCCTGTC | GGGTAAATGGGGAAACAGAGTCAC |
| hnRNPA1 | ATGTCTAAGTCCGAGTCTCCCA | TGTTAGTGTTCCCCATTGCTC |
| CPT1b | ATCATGTATCGCCGCAAACT | CCATCTGGTAGGAGCACATGG |
| CD36 | TGTGTTTGGAGGCATTCTCA | TGGGTTTTGCACATCAAAGA |
| PGC1a | GGACATGTGCAGCCAAGACTCT | CACTTCAATCCACCCAGAAAGCT |
| PPARa | CTCTGTGTGGATGCGGAAGAT | TCCAGACTTCCAACATGAGGA |
| SCD1 | AGGGCAGGTTTCCAAGCGCA | ACTGGAGATCTCTTGGAGCATGTGG |
| TFAM | GGCACAGGAAACCAGTTAGG | CAGAACACCGTGGCTTCTAC |
| NRF1 | CTACTCGTGTGGGACAGCAA | AATTCCGTCGATGGTGAGAG |
| PDK4 | ATCTAACATCGCCAGAATTAAACC | GGAACGTACACAATGTGGATTG |
| SIRT1 | TGTGGTGAAGATCTATGGAGGC | TGTACTTGCTGCAGACGTGGTA |
| ACTIN | GAGACCTTCAACACCCCAGC | ATGTCACGCACGATTTCCC |
